# Differences in Motor and Perceptual Sequence Learning Between People Who Stutter and People Who Do Not Stutter

**DOI:** 10.1002/pchj.70101

**Published:** 2026-05-12

**Authors:** Qianying Ma, Ke Chen, Chen Wang, Meijia Li, Liqun Gao, Lingzhi Kong

**Affiliations:** ^1^ Cognitive Science and Allied Health School Beijing Language and Culture University Beijing China; ^2^ Key Laboratory of Language and Cognitive Science (Ministry of Education), Beijing Language and Culture University Beijing China; ^3^ Psychology Department Vrije Universiteit Brussel and Center for Neuroscience Brussels Belgium; ^4^ Centre for Human Brain Health, School of Psychology University of Birmingham Birmingham UK

**Keywords:** motor learning, perceptual learning, serial reaction time task, stuttering

## Abstract

Previous studies have indicated impaired motor sequence learning in people who stutter (PWS). The present study demonstrates that PWS retain intact sequence learning abilities when motor demands are minimized.

Stuttering is a common communication disorder characterized by involuntary repetitions and prolongations of syllables during speech (Brown et al. 2005). While up to 8% of preschool children aged 2–5 begin stuttering, most recover spontaneously and around one‐fifth persist into adulthood (Neef and Chang 2024).

Sequence learning allows people to acquire complex skills such as speech. Accumulating studies show that people who stutter (PWS) have impaired motor sequence learning performance on the serial reaction time task (SRTT) compared to people who do not stutter (PWNS), as evidenced by longer reaction times (RTs) and slower learning rates (Bauerly and De Nil 2015; Höbler et al. 2022; Neef et al. 2015; Nissen and Bullemer 1987). However, standard SRTT paradigms combine perceptual learning with motor execution, leaving it unclear whether poorer performance in PWS stems from a sequence learning deficit or general motor difficulties. Thus, this study aimed to distinguish whether PWS struggle primarily with sequential motor execution (motor deficit) or with learning sequential regularities (learning deficit).

We recruited 21 PWS and 21 PWNS (Figure 1A). All participants completed both motor and perceptual SRTT. In both tasks, four cartoon animals appeared in a quadrant layout. Participants were asked to respond as fast and as accurately as possible to either “Where is the lion?” (Motor SRTT, Figure 1B) or “Is the unicorn's horn pointed left or right?” (Perceptual SRTT, Figure 1C). Unbeknownst to the participants, a fixed sequence was embedded in the target's locations. The Motor SRTT combined both motor and perceptual learning processes, as the sequence and participants' motor responses were tied to the lion's location. In contrast, the Perceptual SRTT disentangled the learned sequence from motor responses, as the sequence participants had to learn was based on the unicorn's location, while their motor responses were randomly determined by the direction of the unicorn's horn.

**FIGURE 1:**
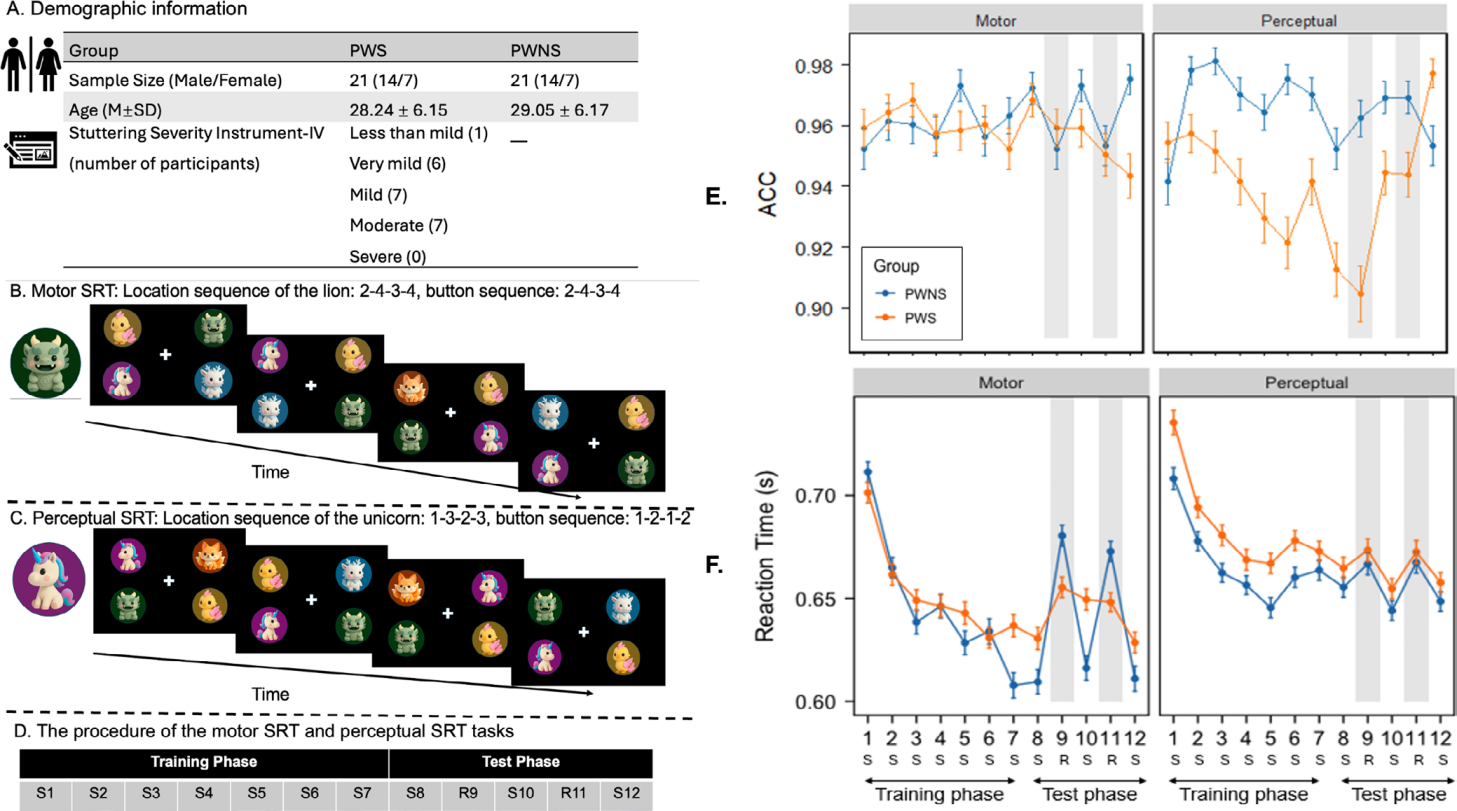
[A]. Demographic information. [B]. Motor SRTT. On each trial, participants reported where the lion was located by pressing “1” to “4” on the keyboard—in this example of four consecutive trials: 2–4–3–4. In the task, the lion's location followed a repeating sequence (2–4–3–4–1–3–2–4). Thus, the sequence to be learned matched the motor response sequence. [C]. Perceptual SRTT. On each trial, participants reported the direction of the unicorn's horn by pressing “1” (left) and “2” (right). In the example, participants should press 1–2–1–2, while unicorn's location followed a repeating sequence (1–3–2–3–4–2–1–3). The direction of unicorn's horn was completely random, hence making the response unpredictable from trial to trial, and dissociating perceptual sequence learning from motor responses. [D]. The SRTT procedure. S = Standard block (with a fixed sequence of target locations). *R* = Random block. [E]. Accuracy across 12 blocks by Group. [F]. Reaction times across 12 blocks by Group. [Left] Motor SRTT. [Right] Perceptual SRTT. Error bars: Standard errors. PWS: People who stutter. PWNS: People who do not stutter.

The SRTT procedure is presented in Figure 1D. The participants first completed the Training phase, consisting of seven 48‐trial Standard blocks (i.e., each containing 6 repetitions of an 8‐trial fixed sequences in one block). This was followed by a Test phase, during which the Standard sequence was occasionally interrupted by a Random block. The Test phase consisted of five 48‐trial blocks.

For data analyses, generalized linear mixed‐effects models (GLMM) were applied using the *lme4* and *lmerTest* packages for R (version 4.3.2). Additionally, the *car* package was used to conduct type III Wald tests, and Tukey corrections were made for multiple comparisons (Firouzi et al. 2024). Specifically, to estimate the effect of stuttering on participants' sequence learning ability, we ran a series of GLMM with Gamma, Gaussian, and Inverse Gaussian families, using a log link. In these models, Group (PWS and PWNS) and Blocks were included as fixed factors. To evaluate general learning effects, we analysed RT improvements across the seven Standard blocks in the Training phase (Blocks S1‐S7). To assess sequence‐specific learning effects, we calculated the different scores (Random—Standard) between averaged RTs across Standard blocks (S8, S10 & S12) and Random blocks (R9 & R11), with larger positive values indicating stronger effects.

As shown in Figure 1E, results revealed high accuracy across both tasks and groups. No group difference was found in Motor SRTT accuracy (*t* = −1.79, *p* = 0.074, *Cohen's d* = 0.19). However, PWS showed significantly lower accuracy than PWNS in the Perceptual SRTT (*t* = −9.97, *p* < 0.001, *Cohen's d* = 0.21).

For RTs in Motor SRTT (Figure 1F left), both PWS and PWNS showed similar RTs at the beginning and improved across the Training phase (*β* = −0.056, *p* < 0.001). Sequence‐specific learning effects showed a significant Group × Block Type interaction (*β* = 0.121, *p* = 0.003). Post hoc analyses revealed that PWNS showed significantly faster RTs in the Standard compared to Random blocks (*β* = −0.141, *p* < 0.001), whereas PWS did not (*β* = 0.020, *p* = 0.479). Overall, PWS showed poorer performance in Motor SRTT than the PWNS.

For RTs in Perceptual SRTT (Figure 1F right), both groups showed significant RT improvements across Standard blocks in the Training phase (all *p* < 0.001), indicating that PWS and PWNS exhibited similar general learning trajectories. Sequence‐specific learning effects were evident in both groups, with faster RTs in the Standard than Random blocks (*β* = −0.029, *p* < 0.001), and no significant Group × Block Type interaction (*β* = 0.003, *p* = 0.804). Thus, both PWS and PWNS showed similar sequence‐specific learning in the perceptual SRTT.

In summary, PWS retain intact learning abilities when motor demands are minimized. That is, PWS showed impaired learning in Motor, not Perceptual SRTT. This dissociation suggests that previously observed impairments in sequence learning may stem from deficits in motor execution rather than the learning process. However, this interpretation remains cautious, as attentional control or strategic adaptation may also influence performance. Additionally, we observed that PWS had comparable accuracy but slower RTs in a complex context with high motor demands. This pattern may reflect a compensatory strategy aimed at preserving task fluency under motor pressure, rather than a deficit in learning efficiency itself. By prioritizing accuracy, PWS may voluntarily choose a speed‐accuracy trade‐off to counterbalance their compromised motor planning and executive networks. Thus, future research should investigate specific strategies applied by PWS across varying motor demands. Clinically, our findings indicate that future protocols for PWS should minimize motor demands, such as providing external coordination through auditory feedback and choral reading, which may enhance learning and speech fluency. Given that PWS showed comparable learning effects to PWNS in Perceptual SRTT, reducing motor demands during interventions may facilitate their acquisition of speech sequences. Meanwhile, achieving fluent performance may reduce the communicative anxiety typically experienced by PWS, and may free up cognitive resources that can be redirected toward the consolidation of learned knowledge. Additionally, treatments should be tailored according to the distinct learning profiles of PWS.

## Funding

This work was supported by the Fundamental Research Funds for the Central Universities of Beijing Language and Culture University, 2442024CXTD02. The Research Funds of Beijing Language and Culture University, 24YCX142.

## Ethics Statement

This study was approved by the Human Research Ethics Committee at Beijing Language and Culture University.

## Consent

All participants gave written informed consent.

## Conflicts of Interest

The authors declare no conflicts of interest.

## Data Availability

The data that support the findings of this study are available from the corresponding author upon reasonable request.
